# Overexpressing lipid raft protein STOML2 modulates the tumor microenvironment via NF-κB signaling in colorectal cancer

**DOI:** 10.1007/s00018-023-05105-y

**Published:** 2024-01-12

**Authors:** Hui Gong, Shaojing Chen, Shuguang Liu, Qianying Hu, Yixuan Li, Yifan Li, Guiqiu Li, Kaimeng Huang, Riqing Li, Lishan Fang

**Affiliations:** 1 Clinical Laboratory, Huazhong University of Science and Technology Union Shenzhen Hospital/Shenzhen Nanshan People’s Hospital/The 6th Affiliated Hospital of Shenzhen University Medical School, Shenzhen, 518052 Guangdong China; 2 Medical Research Center, The Eighth Affiliated Hospital, Sun Yat-Sun University, Shenzhen, 518033 China; 3 Department of Pathology, The Eighth Affiliated Hospital, Sun Yat-Sun University, Shenzhen, 518033 China; 4 Division of Radiation and Genome Stability, Department of Radiation Oncology, Dana-Farber Cancer Institute, Harvard Medical School, Boston, MA 02215 USA; 5 CAS Key Laboratory of Regenerative Biology, Guangzhou Institutes of Biomedicine and Health, Chinese Academy of Sciences, Guangzhou, 510530 China; 6 Shenzhen Agricultural Technology Promotion Center, Shenzhen, 518005 China

**Keywords:** STOML2, Tumor microenvironment, Angiogenesis, Lipid rafts, NF-κB, Colorectal cancer

## Abstract

Colorectal cancer (CRC) is characterized by a complex tumor inflammatory microenvironment, while angiogenesis and immunosuppression frequently occur concomitantly. However, the exact mechanism that controls angiogenesis and immunosuppression in CRC microenvironment remains unclear. Herein, we found that expression levels of lipid raft protein STOML2 were increased in CRC and were associated with advanced disease stage and poor survival outcomes. Intriguingly, we revealed that STOML2 is essential for CRC tumor inflammatory microenvironment, which induces angiogenesis and facilitates tumor immune escape simultaneously both in vitro and in vivo. Moreover, tumors with STOML2 overexpression showed effective response to anti-angiogenesis treatment and immunotherapy in vivo. Mechanistically, STOML2 regulates CRC proliferation, angiogenesis, and immune escape through activated NF-κB signaling pathway via binding to TRADD protein, resulting in upregulation of CCND1, VEGF, and PD-L1. Furthermore, treatment with NF-κB inhibitor dramatically reversed the ability of proliferation and angiogenesis. Clinically, we also observed a strong positive correlation between STOML2 expression and Ki67, CD31, VEGFC and PD-1 of CD8^+^T cell expression. Taken together, our results provided novel insights into the role of STOML2 in CRC inflammatory microenvironment, which may present a therapeutic opportunity for CRC.

**Supplementary Information:**

The online version contains supplementary material available at 10.1007/s00018-023-05105-y.

## Introduction

Colorectal cancer (CRC), one of the most common malignant tumors of the digestive system, has the third-highest incidence rate and the second-highest death rate. Despite the remarkable advances in therapeutic strategies, the 5-year overall survival rate with CRC remains poor [1]. Although, anti-angiogenesis (Bevacizumab) and immunotherapy are used as the first-line treatment and achieve great success in a subset of advanced metastatic CRC patients, it is still of limited efficacy in the majority of cancers [2–5]. Thus, it is crucial to understand the underlying mechanism in order to exploit novel biomarkers and guide CRC therapeutic strategies.

The tumor microenvironment plays an important role in conferring therapeutic resistance by impacting inflammation, angiogenesis, and immune suppression. It has been reported that angiogenesis and immunosuppression frequently and simultaneously occur [6, 7]. Indeed, the combination of anti-angiogenesis therapy and immunotherapy are well-established therapeutic options and exerted synergistic antitumor effects in multiple cancer types [8, 9]. However, it is still unclear about the exact mechanism and key mediators in CRC microenvironment that trigger the concurrence and crosstalk of angiogenesis and immunosuppression.

Lipid rafts are distinct surface areas of cell membranes rich in substances such as cholesterol, sphingolipids, caveolins (CAVs), flotillin-1 (FLOT1), flotillin-2 (FLOT2), stomatin-like protein 2 (STOML2) and other constitutive proteins. They are importantly engaged in a variety of physiological and pathological functions, including tumor development and progression. [10–13]. STOML2, as a critical lipid raft component and a member of the stomatin superfamily, is elevated in gastric, ovarian and cervical cancers, and negatively correlates with the prognosis of patients with cancer. [14–16]. Besides, a recent study has found that in pancreatic cancer STOML2 restricted mitophagy and enhanced chemosensitivity via stabilization of PARL-induced PINK1 degradation [17]. However, the role of STOML2 in modulating CRC tumor microenvironment and progression is poorly understood.

Herein, we found that STOML2 was markedly overexpressed in CRC cell lines and a cohort of human CRC samples and its expression positively associated with advanced clinical stage in CRC. High expression of STOML2 in CRC patients has relatively poor survival rate than those with low expression of STOML2. Interestingly, we showed that STOML2 is crucial for the CRC tumor inflammation microenvironment, which induces proliferation, angiogenesis and immunosuppression in vitro and in vivo. Furthermore, CRC tumors with STOML2 overexpression showed effective response to anti-angiogenesis and immunotherapy. In addition, we found that STOML2 interacted with TRADD protein to activate NF-κB signaling, leading to the upregulation of CCND1, VEGF and PD-L1, while the treatment of NF-κB inhibitor JSH-23 can reverse the ability of proliferation and angiogenesis induced by STOML2 overexpression. Clinically, STOML2 expression was positively correlated with Ki67, CD31, VEGFC and PD-1 of CD8^+^T cell expression. These findings taken together revealed regulatory mechanism of STOML2 and provide evidence for clinical therapeutics.

## Materials and methods

### Cell culture

CRC cell lines, including SW480, SW620, DLD1, HCT116, LoVo and HT-29 and mouse colorectal tumor cells MC38 were purchased from the American Type Culture Collection (ATCC) (Manassas, VA, USA). The normal human colon mucosal epithelial cell lines (NCM460 and FHC) and the CRC cell line (KM12) were purchased from the BeNa Culture Collection (Beijing, China). All cell lines were verified through short tandem repeat (STR) sequence identification. MC38 was grown in RPMI-1640 medium (Invitrogen; Thermo Fisher Scientific); NCM460 and CRC cell lines (SW620, SW480, DLD1, HCT116, KM12, LoVo, and HT-29) were grown in Dulbecco's modified Eagle's medium (DMEM) (Invitrogen) supplemented with 10% fetal bovine serum (Invitrogen) and 100 units of penicillin‒streptomycin (Invitrogen).

### Patient information and tissue specimens

This study was conducted on a total of 119 paraffin-embedded and archived CRC samples, which were diagnosed histopathologically at Huazhong University of Science and Technology Union Shenzhen Hospital from 2003 to 2012. Informed patient consent and approval from the Institutional Research Ethics Committee of Huazhong University of Science and Technology Union Shenzhen Hospital were obtained for use of these clinical materials for research purposes. Clinical information regarding the samples is summarized in Supplemental Table S1.

### Vectors, retroviral infection, and transfection

The polymerase chain reaction (PCR)-amplified human STOML2 coding sequence was subcloned into a pMSCV vector to overexpress STOML2. Two short hairpin RNA (shRNA) oligonucleotides against STOML2 were cloned into the pSuper-retro-puro vector to produce pSuper-retroSTOML2-shRNA(s) to silence endogenous STOML2. The pNF-κB-luc and firefly luciferase-expressing (pRL-TK renilla) plasmids (Clontech) were used to quantitatively assess NF-κB activity. The plasmids were extracted using Endo-free Plasmid Mini Kit (Genebase Bioscience, China). Transfection of plasmids was performed using the Lipofectamine 2000 reagent (Invitrogen).

### RNA extraction, reverse transcription and real-time polymerase chain reaction

Total RNA from CRC cells was extracted using the Total RNA Kit II (Genebase Bioscience, China) following the manufacturer’s instructions. Isolated RNAs were reverse transcribed into cDNA and subsequently quantified in an ABI Prism 7500 Sequence Detection System (Applied Biosystems) using SYBR Green I dye (Molecular Probes, Invitrogen). The primers were synthesized by Sangon Biotech (Supplemental Table S2).

### Western blotting assay

Protein concentration was determined using a BCA assay (Thermo Scientific, USA). Western blotting was performed using anti-STOML2 (CatLog: HPA062016; Sigma-Aldrich), anti-Cyclin D1 (CatLog: SAB5701174; Sigma-Aldrich), anti-VEGFC (CatLog: SAB1411757; Sigma-Aldrich), anti-Flag (CatLog: F7425; Sigma-Aldrich), anti-HA (CatLog: H6908; Sigma-Aldrich), anti-TRADD (CatLog: ab110644; Abcam) antibodies and anti-GAPDH (CatLog: ab8245; Abcam) antibody as a loading control.

### Immunohistochemistry (IHC)

IHC was conducted to analyze expression levels in 50 human formalin-fixed and paraffin-embedded (FFPE) CRC tissues. The degree of immunostaining of FFPE sections was based on both the proportion of positively stained tumor cells and the intensity of immunostaining, which was assessed and scored independently by two observers. The stained sections were evaluated at 400 × magnification, and 10 staining fields of each section were selected randomly and analyzed. The proportion of positive tumor cells was counted and scored as follows: 0 (< 25%); 1 (25–50%); 2 (50–75%) and 3 (> 75%). The intensity of staining was scored as follows: 0 (no staining); 1 (light yellow); 2 (yellow brown) and 3 (brown). The staining index (SI) was calculated as the proportion of positive tumor cells × staining intensity score. Low expression was defined as an SI of 0–3 points, and strong expression was defined as an SI > 3 points.

### Colony formation assay

In 6-well plates, CRC cells were seeded with 300 cells and grown for 10 days. After being fixed with 10% formaldehyde for 5 min, the colonies were stained with 1% crystal violet for 30 s.

### Tube formation assay in human umbilical vein endothelial cells (HUVECs)

For the tube formation assay, 200 μL Matrigel was first added into each well of a 24-well plate and polymerized for 30 min at 37 °C. Next, 4 × 10^4^ HUVECs were added to the precoated Matrigel, cultured with conditioned medium from the indicated CRC cells and incubated at 37 °C in 5% CO_2_ for 20 h. The capillary tubes were measured by tallying their length after images were captured using a 100 × bright-field microscope.

### Flow cytometry

The cell surface PD‐L1 expression in the cells were detected by flow cytometry. Cells following various treatments were gently trypsinized and collected by centrifugation. After washed twice with PBS, cells were stained with an anti-PD-L1 antibody (ABF133; Sigma-Aldrich) and anti-STOML2 antibody (ab191883, Abcam). To quantify PD-1 expression on CD8^+^ T cells, whole blood into an EDTA tube was collected and subjected to red blood cell lysis followed by and collected by centrifugation. Cell pellets were subsequently stained with different antibodies for flow cytometry analysis. Cell populations were discriminated by the following antibodies: anti-CD45 (SAB4700587; Sigma-Aldrich), anti-CD3 (SAB4700044; Sigma-Aldrich), anti-CD8 (SAB4700084; Sigma-Aldrich), and anti-PD1 (SAB5701115; Sigma-Aldrich). Cell populations and marker expression were gated and analyzed using the FlowJo software: leukocytes (CD45^+^), T lymphocytes (CD45^+^CD3^+^), and CD8^+^ T cells (CD45^+^CD3^+^CD8^+^).

### Luciferase assay

Cancer-related pathways analysis was performed using the Cignal Finder TM 10-Pathway Reporter Arrays (Qiagen, Dusseldorf, Germany) according to the manufacturer’s instruction. In 48-well plates, 3000 cells were plated in each well in triplicate and cultured for 24 h. Using the Lipofectamine2000 reagent (Invitrogen), 100 ng of luciferase-expressing plasmids of the pNF-κB-luc reporter along with 1 ng of pRL-TK renilla plasmid were transfected into vector-control of STOML2-overexpressing or -silenced CRC cells in accordance with the manufacturer’s instructions. The Dual Luciferase Reporter Assay Kit (Promega) was used to detect the luciferase and renilla signals 24 h after transfection.

### Immunofluorescence assay

SW480 cells grown on coverslips were incubated with anti-CTxB antibody (SAB4200844, Sigma-Aldrich) and secondary antibody conjugated with Alexa Fluor 488. Nuclei were counterstained with DAPI. The images were acquired on a Zeiss LSM780 confocal microscope, and an optimum voxel size was determined by Zeiss Zen software.

### Animal experiments

All studies involving C57BL/6 and BALB/c nude mice were approved by the Institutional Animal Care and Use Committee of Shenzhen University. Sample sizes were determined by power analysis during the animal ethics dossier application. At least five mice per group were used to ensure the adequate power and each mouse with different weight was randomly allocated. For MC38 syngeneic tumor model, Stoml2-overexpressing, Stoml2-silenced MC38 cells or corresponding control (1 × 10^6^) were subcutaneously injected into the dorsal flanks of female C57BL/6 (6–8 weeks old, 20-23 g) mice. On day 40 after inoculation, the mice were killed, and the tumors were excised, weighed and paraffin-embedded. Serial 6.0 μm sections were sliced and subjected to IHC analysis using anti-Ki67, CD31 and PD-L1 antibodies (Dako). For the immunotherapy evaluation experiment, MC38-Stoml2-overexpressing or MC38-vector tumor-bearing female C57BL/6 mice were divided randomly into control (IgG, BioXcell, Clone 2A3) and treatment groups (anti-PD1, BioXcell, Clone RMP1-14). For the bevacizumab administration experiment, the female BALB/c nude mice (4–5 weeks old, 18–20 g) bearing STOML2-overexpressing, STOML2-silenced or control HCT116 tumor xenografts (n = 5/group) were intravenously injected with control solvent and bevacizumab twice a week for 4 consecutive weeks. Resultant tumors were examined and measured by length and width using calipers twice weekly, and tumor volumes were calculated using the formula (width2*length)/2.

### Data acquisition

Gene expression data used for comparison of STOML2 gene expression between cancerous and normal tissues in CRC from The Cancer Genome Atlas (TCGA) were downloaded from the UCSC XENA (https://xenabrowser.net/). The GSE8671, GSE21510, and GSE20916 datasets were obtained from Gene Expression Omnibus (GEO, http://www.ncbi.nlm.nih.gov/geo). Protein abundance data of STOML2 expression levels between cancerous and normal tissues in CRC were obtained from Proteomic Data Commons (PDC, https://pdc.cancer.gov/pdc/).

### Statistical analysis

All statistical analyses were performed with the SPSS 19.0 statistical software package. *Χ*^2^ test was performed to analyze the correlation between STOML2 expression and clinicopathological characteristics. The survival curve was established by the Kaplan –Meier method and compared by the log-rank test. The Cox regression model was employed to conduct univariate and multivariate analysis. Two-tailed Student’s t test was used to compare statistical significance between two groups. Sample size was determined by power analysis to achieve a minimum effect size of 0.5 with a *p* value of < 0.05 and all sample sizes were appropriate for assumption of normal distribution. Variance within each group of data was estimated and was similar between compared groups. All experiments were performed in triplicate (*p < 0.05; **p< 0.01; ns, non-significant).

## Results

### Increased expression of STOML2 correlates with CRC progression and poor prognosis

**Fig. 1:**
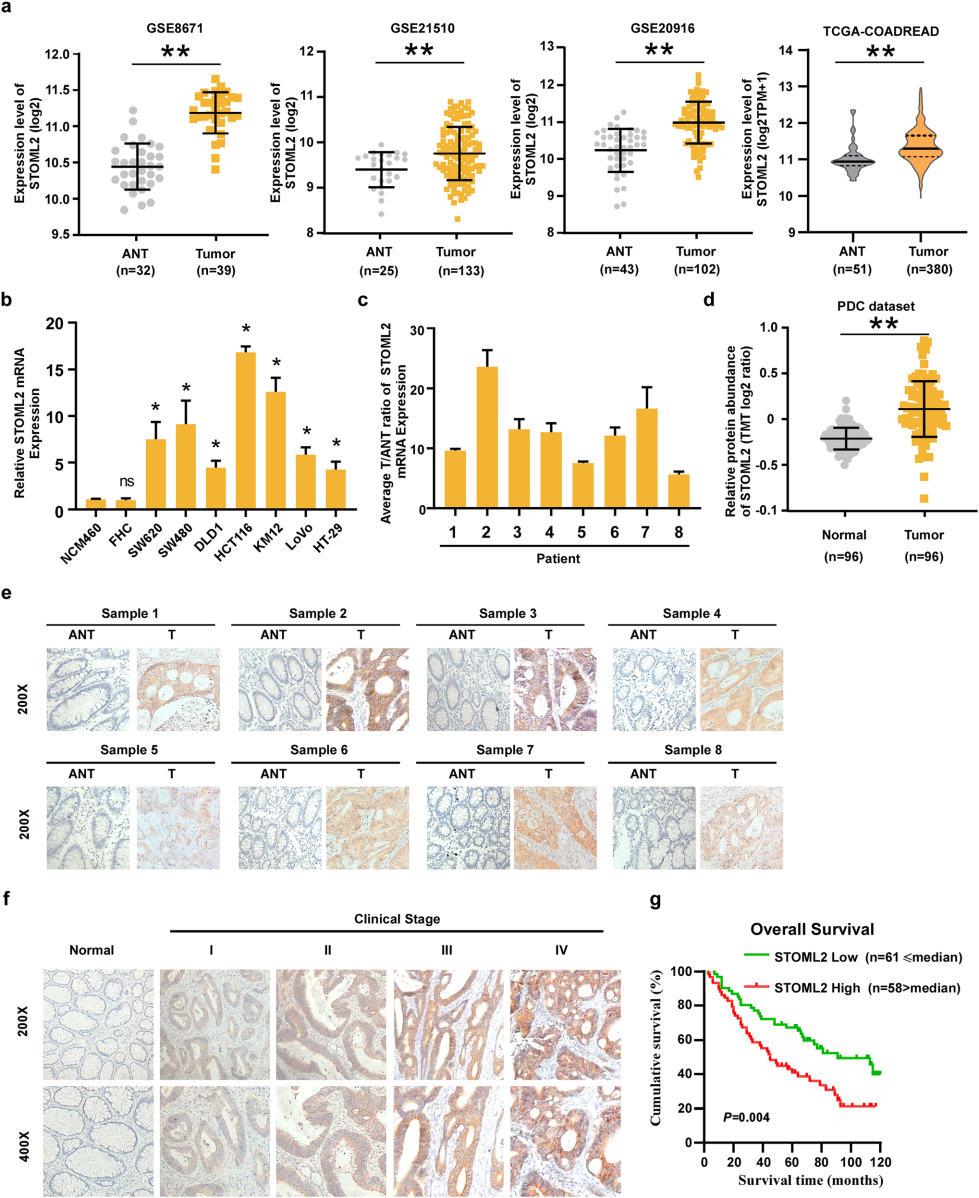
STOML2 is upregulated in CRC cell lines and CRC tissues. **a** Relative expression levels of STOML2 in CRC and normal tissues analyzed using the GSE8671 GSE21510, GSE20916 and TCGA datasets. **b** STOML2 mRNA expression in 2 normal colonic epithelial cell lines (NCM460 and FHC) and 7 CRC cell lines. **c** Real-time PCR analysis of STOML2 mRNA in 8 paired primary CRC tissues (T) and adjacent noncancerous tissues (ANT) from the same patient. Expression levels were normalized to GAPDH. **d** Relative protein abundance of STOML2 in CRC and pair matched normal tissues from the PDC dataset. **e** IHC staining of STOML2 in 8 pairs of CRC tissue and adjacent nontumor tissue. **f** IHC staining of STOML2 in stage I–IV CRC tissues and normal tissues. **g** Kaplan‒Meier analysis of overall survival for STOML2 expression in CRC patients

To explore the importance of STOML2 in CRC progression, we first analyzed the expression level of STOML2 in CRC and adjacent noncancerous tissues (ANTs) from public datasets. As shown in Fig. 1a, the STOML2 mRNA level was significantly upregulated in CRC tissues compared with ANTs from the TCGA and GSE8671, GSE21510, GSE20916 datasets. When compared with normal colonic epithelial cells (NCM460 and FHC), STOML2 was upregulated in all these CRC cell lines (Fig. 1b). Consistently, we also observed a significant increase of STOML2 expression in 8 human CRC samples relative to their paired noncancerous adjacent colon tissues (Fig. 1c, e). Moreover, analysis of 96 pairs of CRC samples from the PDC dataset showed elevated protein abundance of STOML2 in CRC tissues when compared with matched ANTs (Fig. 1d). Collectively, these results strongly suggest that STOML2 is highly expressed in CRCs.

The observed overexpression of STOML2 in CRC prompted us to further investigate the clinical relevance of STOML2 in the progression of CRC. We therefore extended our STOML2 quantification experiment to a cohort of 119 archived paraffin-embedded specimens of CRC, and the level of STOML2 expression closely associated with CRC clinical staging (*p* < 0.001), TNM classification (*p* = 0.003,  *p* 0.001, and *p* = 0.048, respectively), and histological differentiation (*p* = 0.016) but did not correlate with gender, age and tumor localization (Table 1). As shown in Fig. 1f, STOML2 expression, which was barely detectable in normal colorectal tissue, was significantly elevated in advanced-stage (stages III and IV) CRC tissues as compared to early-stage (stages I and II) CRC tissues. Furthermore, Kaplan–Meier analysis using the log-rank test was conducted and the result showed that patients with low STOML2 expression had a longer median survival time of 68 months than those with high STOML2 expression, whose median survival time was 45 months (*P* = 0.004; Fig. 1g) indicating a worse prognosis outcome for CRC patients with high STOML2 expression. To better understand how STOML2 contribute to CRC progression, we performed both univariate and multivariate Cox regression analyses of clinicopathological feature parameters and STOML2 expression in CRC patients. In the univariate Cox analysis, STOML2 expression, T stage and M stage were valuable prognostic factors. In the multivariate Cox regression analysis, only STOML2 expression and M stage served as independent prognostic factors (Table 2). Collectively, these data suggest that increased STOML2 expression might contribute to CRC progression.

**Table 1:** Correlation between the clinicopathological features and expression of STOML2

Patient characteristics	STOML2 expression		*P*-value
	Low or none	High	
Gender			0.715
Male	34	30	
Female	27	28	
Age (years)			0.534
≤ 62	31	30	
> 62	30	28	
Tumor location			0.302
Ascending colon	11	6	
Transverse colon	9	4	
Ascending colon	6	11	
Sigmoid colon	7	7	
Rectal	28	30	
Clinical stage			0.001
I	15	2	
II	20	13	
III	17	25	
IV	9	18	
T classification			0.003
T_1_	2	0	
T_2_	20	7	
T_3_	22	19	
T_4_	17	32	
N classification			0.001
N_0_	40	19	
N_1_	14	23	
N_2_	7	16	
M classification			0.048
No	52	40	
Yes	9	18	
Histological differentiation			0.016
Well	21	13	
Moderate	25	16	
Poor	15	29

**Table 2:** Univariate and multivariate analysis of different prognostic parameters in patients with CRC by Cox-regression analysis

	Univariate analysis			Multivariate analysis		
	No. patients	*P*	Regression coefficient (SE)	*P*	Relative risk	95% confidence interval
T classification		0.017	0.148			
T_1_	2					
T_2_	27					
T_3_	41					
T_4_	49					
M classification		< 0.001	0.268	< 0.001	2.975	1.742–5.082
M_0_	92					
M_1_	27					
Expression of STOML2		0.005	0.243	0.040	1.670	1.024–2.724
Low expression	61					
High expression	58					

### STOML2 promotes the aggressiveness of CRC Cells in vitro

**Fig. 2:**
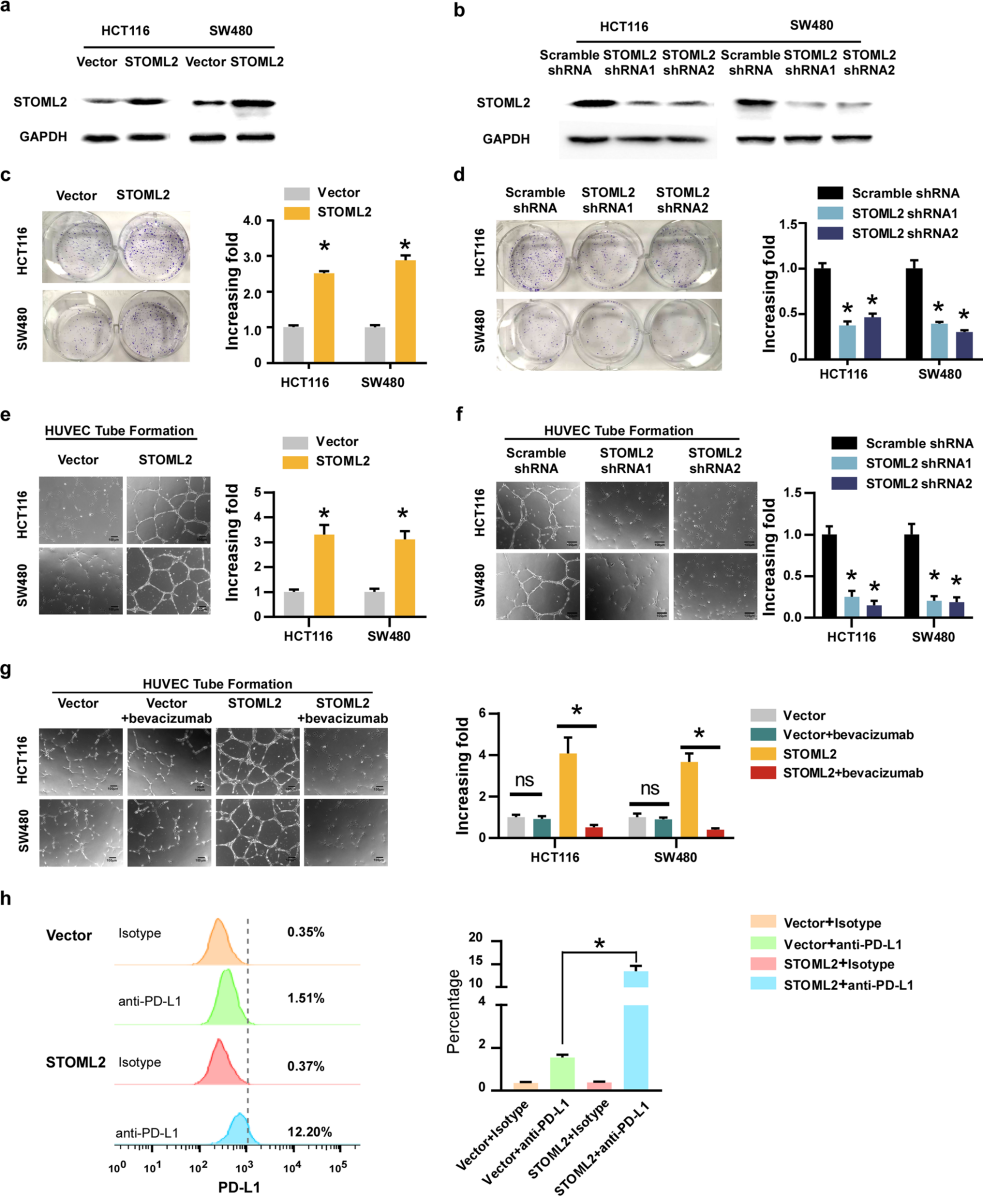
STOML2 promoted CRC progression in vitro. **a**, **b** Western blot analysis of STOML2 expression levels in HCT116 and SW480 cells. GAPDH was used as a loading control. **c**, **d** Colony formation of STOML2-overexpressing or STOML2-silenced CRC cells. **e**, **f** Effects of STOML2 on tube formation assay with HUVECs. **g** Effects of bevacizumab treatment on the tube formation assay with HUVECs stimulated with conditioned medium from indicated cells. **h** The expression of PD-L1 on the cell membrane in SW480-STOML2 -overexpressing or empty vector-transducted cells

To investigate whether STOML2 plays a role in the pathogenesis of CRC, we performed gain and loss of function of STOML2 in two CRC cells lines. Overexpression and silencing of STOML2 was confirmed by western blot (Fig. 2a, b). Colony formation assays showed that STOML2 overexpression remarkably promoted, whereas silencing of STOML2 inhibited the growth of CRC cells (Fig. 2c, d). Moreover, the overexpression of STOML2 significantly enhanced, whereas silencing STOML2 strongly compromised the ability of CRC cells to induce tubule formation of human umbilical vein endothelial cells (HUVECs) (Fig. 2e, f), suggesting STOML2 might be involved in the angiogenesis of CRC cells. Moreover, Bevacizumab treatment potently eliminated the capacity of STOML2-overexpressing induced HUVEC tubule formation, while it has no additional effect in the STOML2-silenced cells (Fig. 2g, Supplemental Fig. 1).

Angiogenesis and immunosuppression frequently occur concomitantly [6], we therefore investigate whether the role of STOML2 in CRC immunosuppression. Since Programmed Cell Death Ligand 1 (PD-L1) is an important negative-regulatory ligands for immune response[11], we conducted the flow cytometry analysis and surprisingly found that overexpression of STOML2 upregulated PD-L1 expression (Fig. 2h), indicating STOML2 could also activate the immune suppression response in CRCs. Taken together, these data demonstrate that STOML2 plays a prominent oncogenic role in promoting CRC cell proliferation, angiogenesis and immune escape.

### STOML2 contributes to the progression of CRC in vivo

**Fig. 3:**
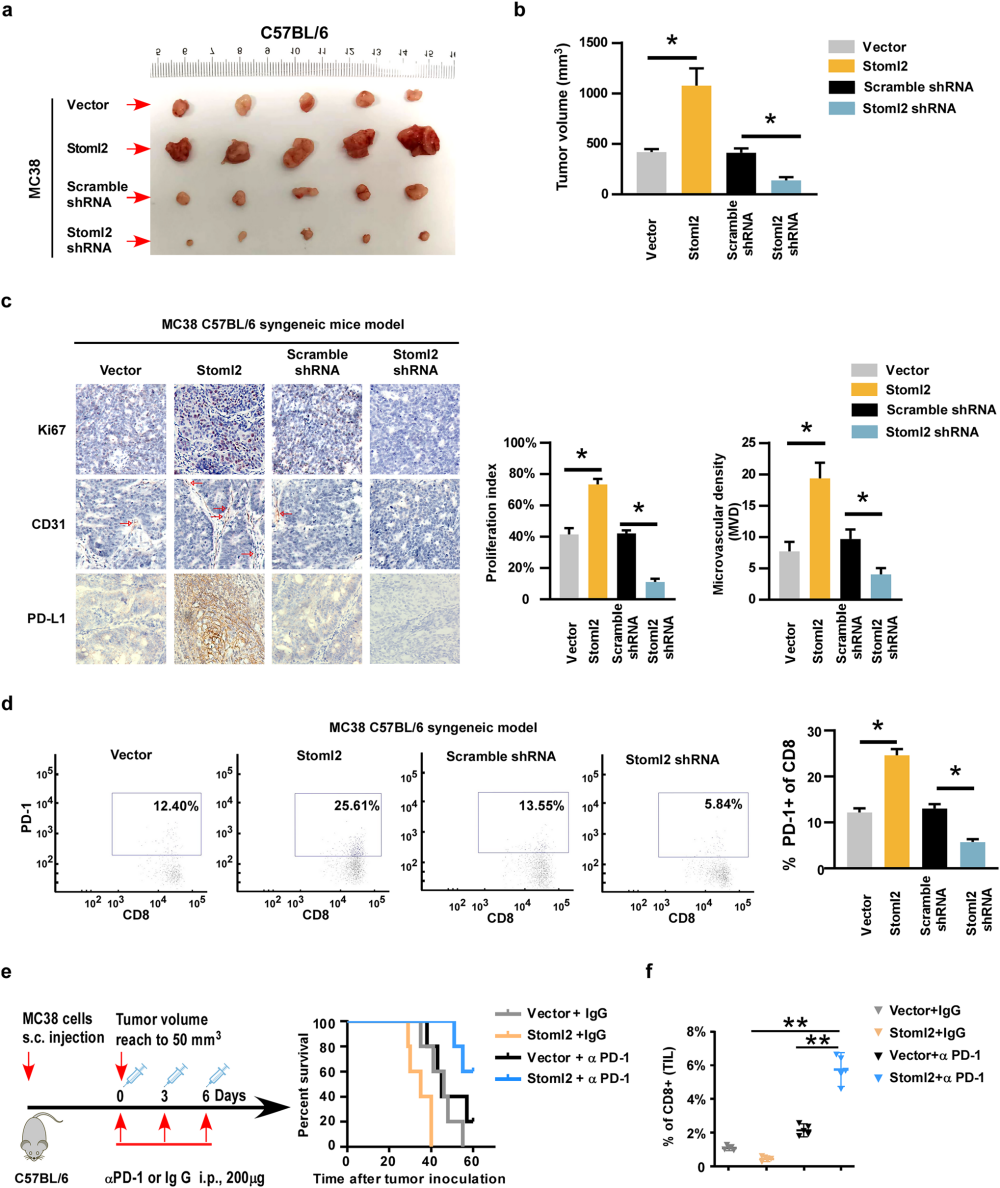
STOML2 enhanced the aggressive phenotype of CRC in vivo. **a** Representative images of tumors formed by the indicated cells on C57BL/6-MC38 syngeneic model. **b** Quantitative analysis of tumor volume (right pannel). **c** IHC staining showed the levels of Ki67, CD31 and PD-L1 in overexpressing or silencing STOML2 tumor. The bar graph shows the Ki67 proliferation index and microvascular density (MVD, marked by CD31). **d** Representative flow cytometry plots of Pd-1 expression on CD8^+^ T cells in the indicated MC38 syngeneic tumor model. **e** Schematic diagram depicting the treatment of C57BL/6-MC38 syngeneic model with either anti-PD1 antibody or IgG isotype. Kaplan–Meier survival curves for indicated treatment group (right pannel). **f** The tumor infiltrated Cd8^+^ T cells of indicted treatment group and presented as mean values ± SD (*n* = 5)

Our in vitro data prompted us to investigate whether STOML2 could also function similarly in vivo. To answer the question, MC38 cells were injected into immunocompetent C57BL/6 mice to establish the subcutaneous tumor model. Consistent with in vitro result, tumors derived from MC38 Stoml2-overexpressing was significantly larger than those induced by vector control, whereas Stoml2 silencing had a significant attenuated ability to form tumors as compared to scramble shRNA (Fig. 3a, b). Furthermore, Ki67-positive proliferation index, microvascular density (MVD), PD-L1 expression were significantly increased in Stoml2-overexpressing tumor and reduced in the Stoml2-silenced tumor as compared to their respective counterparts (Fig. 3c). In addition, mice with Stoml2 overexpression showed higher ratio of the Pd-1^+^ on Cd8^+^, while mice bearing Stoml2-silenced tumor exhibited lower ratio of the Pd-1^+^ on Cd8^+^ compared to their corresponding controls (Fig. 3d).

Given the role of STOML2 in modulating the expression PD-L1 in colorectal cancer cells, we next assessed the potential benefit of Stoml2 in tumor treated with immune checkpoint blockage (ICB). The immunocompetent C57BL/6 mice bearing Stoml2-overexpressing or control tumor were treated with either anti-PD-1 antibody or IgG control antibody. Intriguingly, anti-PD-1 treatment strongly prolonged the survival time and increased the tumor infiltration of Cd8^+^ T cells in the Stoml2-overexpressing group, while immunotherapy had a limited effect on the mice survival and moderately increased the proportion of infiltration Cd8^+^ T cells (Fig. 3e, f).

**Fig. 4:**
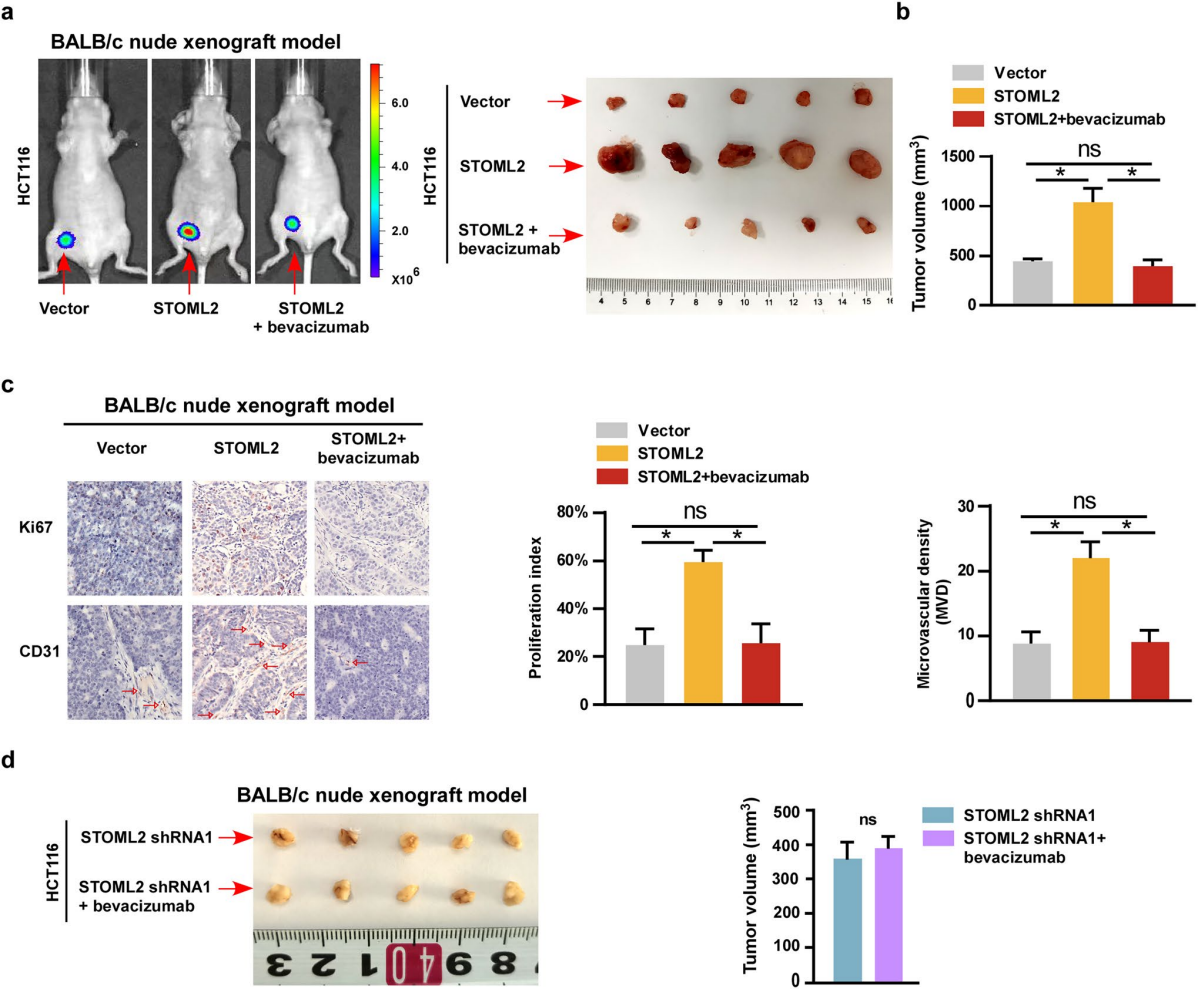
STOML2 induced CRC progression inhibited by bevacizumab treatment. **a** Representative pictures of tumor-bearing mice and xenograft tumors in BALB/c nude mice HCT116-xenograft model. **b** Quantitative analysis of tumor volume showing the effects of bevacizumab treatment in BALB/c nude mice HCT116-xenograft model. **c** IHC staining showed the levels of Ki67 and CD31 with or without bevacizumab treatment. The bar graph showed the Ki67 proliferation index and microvascular density. **d** The representative images of xenograft and tumor volume formed by STOML2-silenced cells treated with or without bevacizumab

To investigate the role of STOML2 in response to anti-angiogenic treatment in vivo, immunodeficient BALB/c nude mice bearing STOML2-overexpressing, STOML2-silenced or control HCT116 tumor xenografts were treated with control solvent or bevacizumab antibody. As shown in Fig. 4a, b, bevacizumab treatment impressively inhibited the tumor growth rate and volume induced by STOML2-overexpression. Consistently, Ki67 and CD31 expression were markedly reduced by bevacizumab treatment in STOML2-overexpressing xenografts (Fig. 4c). Of note, in line with the in vitro experiments, bevacizumab treatment has no additional effect in tumors derived from STOML2-silenced HCT116 xenografts (Fig. 4d).

### STOML2 promotes CRC progression via NF-κB signaling pathway

**Fig. 5:**
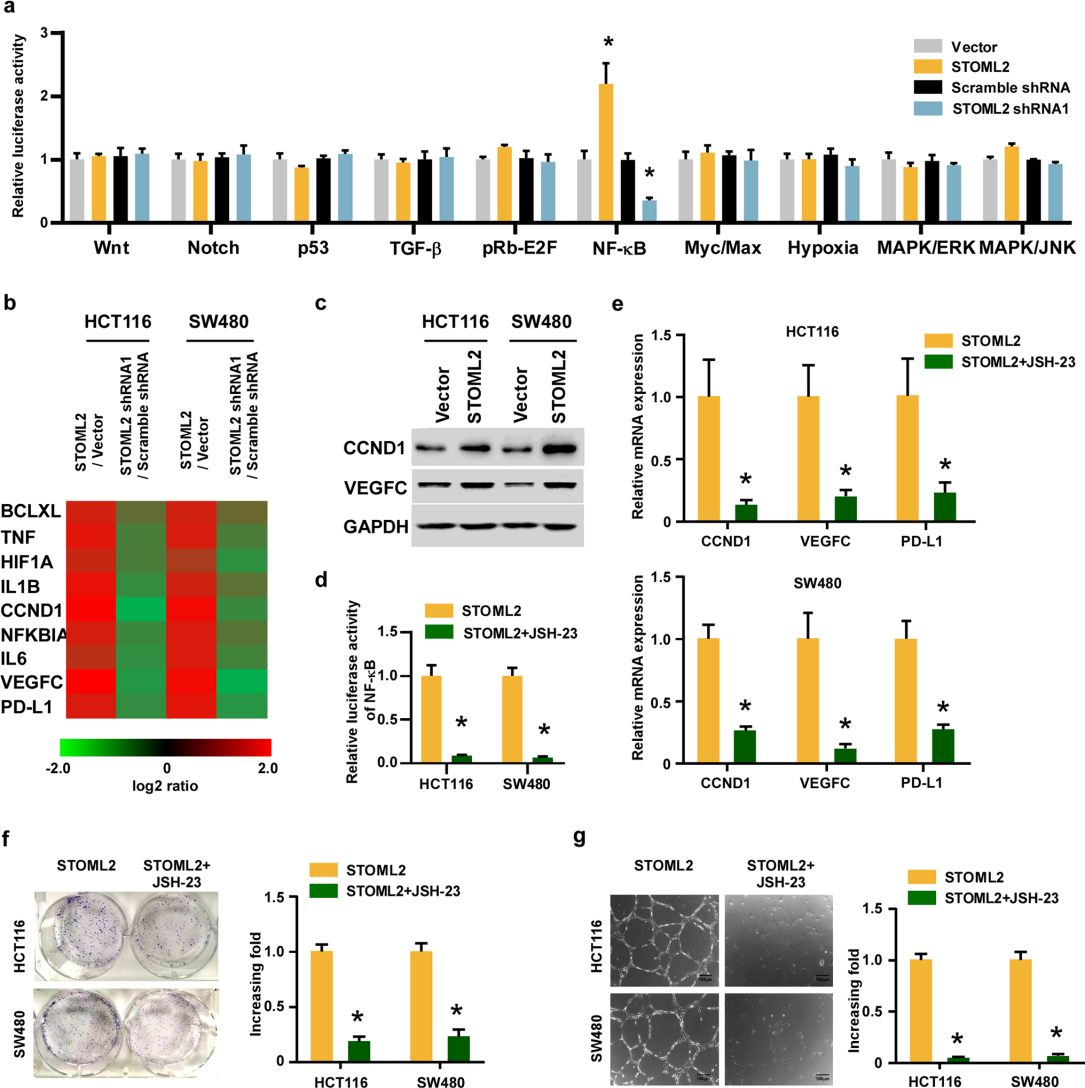
STOML2 activates the NF-κB signaling pathway. **a** The luciferase reporter activity of cancer-associated pathways was detected in the indicated cells. **b** RT-q-PCR assay indicated an apparent overlap between NF-κB-dependent gene expression and STOML2-regulated gene expression. **c** Western blot analysis of Cyclin D1 (CCND1) and VEGFC expression levels in the indicated cell lines. GAPDH was used as a loading control. **d** The luciferase reporter activity of NF-κB was detected in the indicated CRC cells treated with or without NF-κB inhibitor, JSH-23. **e** The mRNA expression levels of CCND1, VEGFC and PD-L1 in HCT116 and SW480 cells treated with or without JSH-23. **f** Representative images and quantification of colony formation assays on STOML2-overexpressing CRC cell lines treated with or without JSH-23. **g** Representative images and quantification of HUVECs stimulated by the cluture medium of the indicated cell lines treated with or without JSH-23

To decipher the regulatory mechanisms of STOML2 in CRC progression, we conducted the Cignal Finder Reporter Arrays for the comprehensive screening to pinpoint the pathways. Prominently, we found that overexpressing STOML2 significantly enhanced, whereas silencing STOML2 repressed NF-κB luciferase activity, as well as the expression levels of numerous well-known NF-κB downstream genes, compared with the respective control (Fig. 5a, b; Supplemental Fig. 2). Specifically, the expression levels of tumor proliferation and angiogenesis protein CCND1 and VEGFC were upregulated by STOML2 overexpression (Fig. 5c). To validate that STOML2 mediated-CRC pathogenesis was through NF-κB activation, we then examined the effect of blocking the NF-κB pathway on STOML2-induced aggressiveness. As expected, the stimulatory effect of STOML2 on NF-κB activation, the upregulation of CCND1, VEGFC and PD-L1 expression was remarkable inhibited by NF-κB inhibitor JSH-23 (Fig. 5d, e). Furthermore, the ability of STOML2-induced tumor growth and HUVEC tube formation was also strongly reversed by the JSH-23 (Fig. 5f, g). Collectively, these results indicate that activation of NF-κB is critical for the oncogenic function of STOML2.

**Fig. 6:**
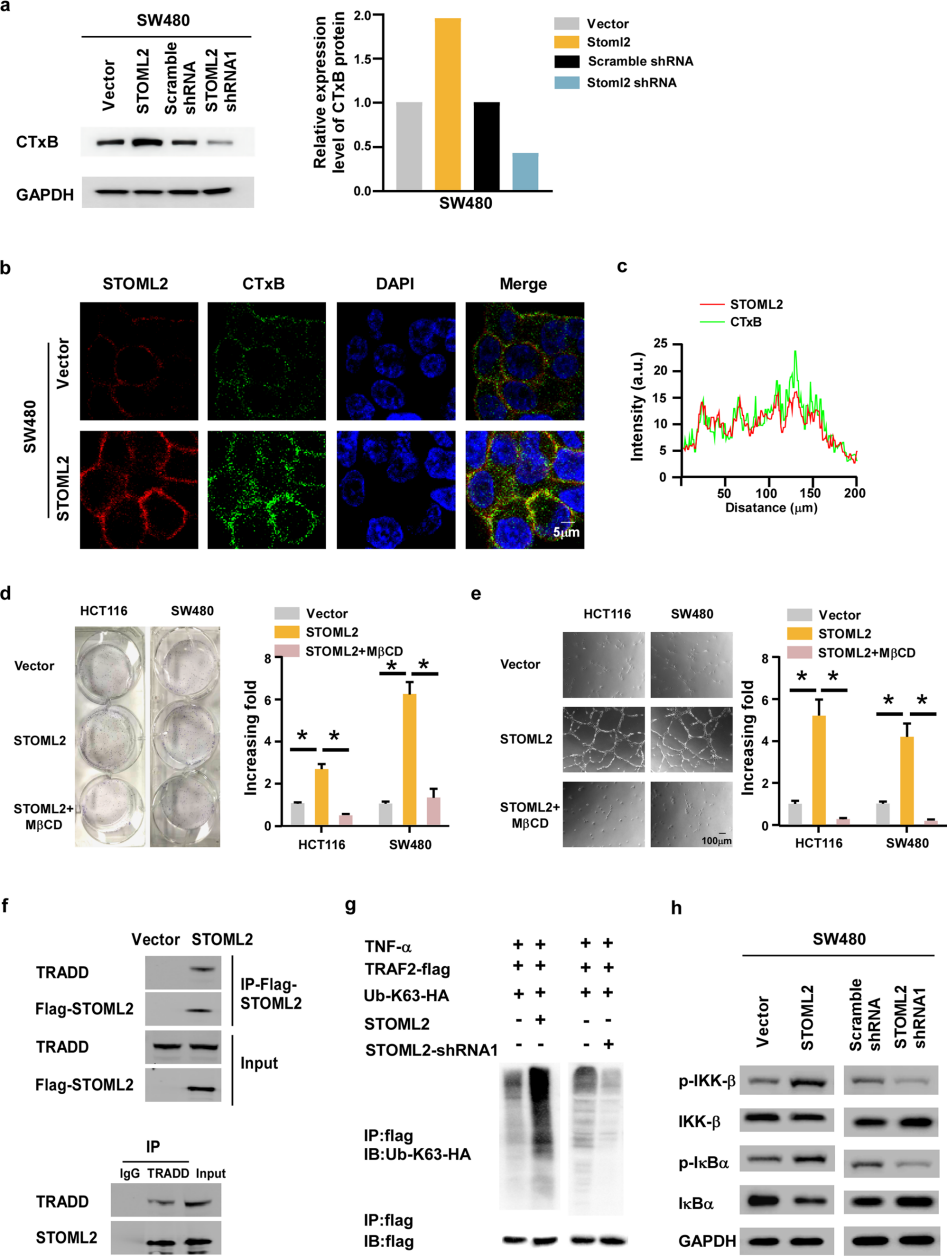
STOML2 interacted with TRADD protein to activate NF-κB signaling pathway. **a** Western blot analysis of lipid rafts marker CTxB expression levels in the indicated cell lines. **b** Immunostaining of STOML2 and CTxB in the indicated cell lines. **c** Fluorescence intensity and colocalization of STOML2 and CTxB on cell membrane. **d** Representative images and quantification of colony formation assays in indicated cell lines treated with or without the methyl-β-cyclodextrin (MβCD). **e** Representative images and quantification of HUVECs cultured with conditioned medium from the indicated CRC cells treated with or without MβCD. **f** Immunoprecipitates and corresponding total cell lysates were subjected to western blotting using STOML2 or TRADD antibodies. **g** Western blot analysis of K63-linked polyubiquitin of TRAF2 in indicated cells treated with TNF-α (10 ng/mL). **h** The expression levels of IKK-β, IκBα and corresponding phosphorylated protein in STOML2-overexpressing or -silenced SW480 cells

More importantly, Western blot analysis and immunofluorescence staining revealed that the quantity of the ganglioside GM1 dramatically increased in STOML2-transduced cells and decreased in STOML2 knockdown cells compared with vector cells, identified by cholera toxin subunit B (CTxB), a marker of lipid rafts (Fig. 6a). Furthermore, we also observed colocalization of STOML2 with lipid raft marker on the cell membrane (Fig. 6b, c), indicating that STOML2 plays an essential role in the formation of lipid rafts. In addition, disrupting lipid rafts using methyl-β-cyclodextrin (MβCD) significantly attenuated the ability of STOML2-induced NF-κB activation, proliferation and angiogenesis (Fig. 6d, e), suggesting that lipid rafts are essential for STOML2-mediated NF-κB activation.

Since the recruitment of Tumor Necrosis Factor Receptor-Associated Death Domain (TRADD) to lipid rafts is essential for the formation of TRADD-RIP-TRAF2 complex and initiates NF-κB activation [18], immunoprecipitation assays were conducted and the result showed that STOML2 coprecipitated with TRADD via a physical interaction (Fig. 6f). Consistently, K63-polyubiquitination level of TRAF2, as well as the expression levels of phosphorylated-IKK-β and-IκBα, were stimulated by STOML2-transducted, and dramatically reduced when STOML2 silenced (Fig. 6g, h), suggesting that STOML2 promotes the ubiquitin conjugation of NF-κB signaling and sustains NF-κB activity.

### Clinical relevance of STOML2-induced NF-κB activation in clinical CRC tissues

**Fig. 7:**
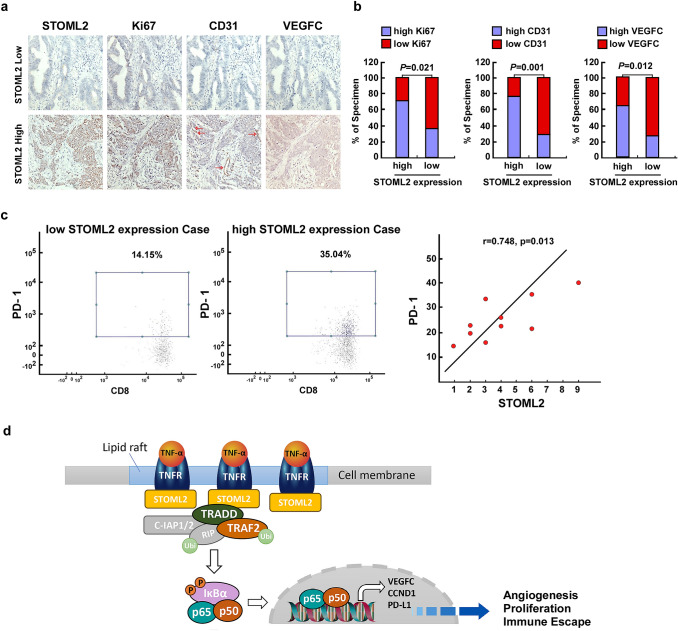
Clinical relevance of STOML2-induced NF-κB activation in CRC. **a** STOML2, Ki67, CD31 and VEGFC levels in 50 CRC specimens. The two representative specimens exhibited low and high expression of STOML2. **b** Percentage of specimens showing high- and low expression of Ki67, CD31 and VEGFC in patient specimens with high and low STOML2 expression. **c** Representative flow cytometry plots of PD-1 expression on CD8^+^T cells (CD45^+^ CD3^+^ CD8^+^) in patient specimens with high and low STOML2 expression. Correlation analysis of STOML2 expression and the expression level of PD-1 in CD8^+^ T cells. **d** A flowchart to show how STOML2 activate the NF-κB signaling pathway

To examine the clinical relevance of STOML2 and the NF-κB pathway in CRC, we analyzed the correlation of STOML2 expression with Ki67, CD31 and VEGFC expression in 50 collected human CRC specimens. As shown in Fig. 7a, STOML2 levels were strongly correlated with the expression of Ki67, CD31 and VEGFC. In the high STOML2 expression group, 71.4%, 75.0% and 64.3% of CRC specimens showed high levels of Ki67, CD31 and VEGFC, respectively, whereas 63.6%, 72.7% and 72.7% of specimens with low STOML2 expression exhibited low expression of Ki67, CD31 and VEGFC, respectively (Fig. 7b). Furthermore, we also found that PD-1 expression on CD8^+^ T cells was positively correlated with STOML2 expression (Fig. 7c). Collectively, our findings indicate that STOML2 facilitates the recruitment of TRADD and sustains NF-κB activity to upregulate CCND1, VEGFC and PD-L1, which consequently leads to tumor proliferation, angiogenesis, immune escape and poorer clinical outcomes in human in CRC (Fig. 7d).

## Discussion

STOML2 has been reported to closely related to the occurrence and progression of various tumors, and overexpression of STOML2 is associated to poor prognosis in several cancers, including CRC [19, 20]. Thus, the role of STOML2 in tumor progression received widespread attention. In the present study, we found that STOML2 is highly expressed in CRC and simultaneously foster neovascularization and immune escape by activating the NF-κB signaling pathway. Collectively, STOML2 fundamentally crucial in CRC tumorigenesis, which may provide new insight into the therapy strategy for CRC.

Currently, neovascularization, immune evasion are the hallmarks of CRC microenvironment. The antiangiogenic agent bevacizumab has been widely implicated in targeted therapy of tumors, including CRC [21]. In our study, we demonstrated that the lipid raft protein STOML2 upregulates VEGFC expression to promote tumor angiogenesis, leading to the increased sensitivity to bevacizumab. Besides that, immunotherapy is a promising treatment for CRC, but the success rates are limited because of the presence of de novo and acquired resistance [22]. Our subsequent experiments revealed that STOML2 triggers the upregulation of PD-L1 expression level and mediates the tumor immune escape indicating that STOML2 contributed to CRC progression via promoting the concurrent of angiogenesis and immunosuppression.

The previous studies suggested that NF-κB can be recruited to lipid rafts and subsequently activated, which plays a key role during tumor development and progression [23–25]. For example, TNFR is translocated into lipid rafts in response to TNF-α stimulation and leads to NF-κB signaling activation [18]. In addition, NF-κB has been reported to regulate the expression of PD-L1, which might contribute to an immune suppressive environment [26]. Studies have also shown that suppression of STOML2 reduces IL-6 expression in glioma by inhibiting the transcription of NF-κB [27]. In our study, we showed that STOML2 overexpression increased NF-κB activity, which drove the expression of CCND1, VEGF and PD-L1 via binding to TRADD protein.

Collectively, our results for the first time demonstrate that STOML2 is up-regulated and indicated an unfavorable prognosis in CRC by activating NF-κB signaling pathway and modulating cell proliferation, angiogenesis and immune evasion, which provide a promise for diagnosis and treatment for CRC.

### Supplementary Information

Below is the link to the electronic supplementary material.

Supplementary file1 (DOC 564 KB)

## Data Availability

The data generated in this study are available upon request from the corresponding author.
