# Supplementary material for: Overexpressing lipid raft protein STOML2 modulates the tumor microenvironment via NF-κB signaling in colorectal cancer
Source: Cell Mol Life Sci. 2024 Jan 12;81(1):39. doi: 10.1007/s00018-023-05105-y (PMC10786741; doi:10.1007/s00018-023-05105-y)
Supplement: Supplementary file 1 — Supplementary file1 (DOC 564 KB) [file 18_2023_5105_MOESM1_ESM.doc]

**Supplemental Figures**

**Supplemental Figure 1**


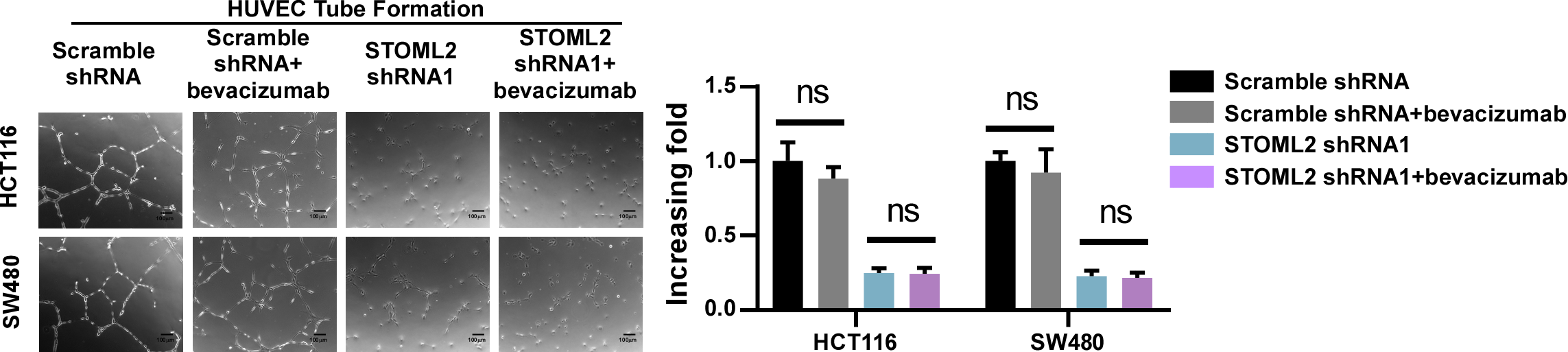


Supplemental Figure 1 Effects of bevacizumab treatment on the tube formation assay with HUVECs cultured with conditioned medium from the STOML2-silencing or control CRC cells.

**Supplemental Figure 2**

**
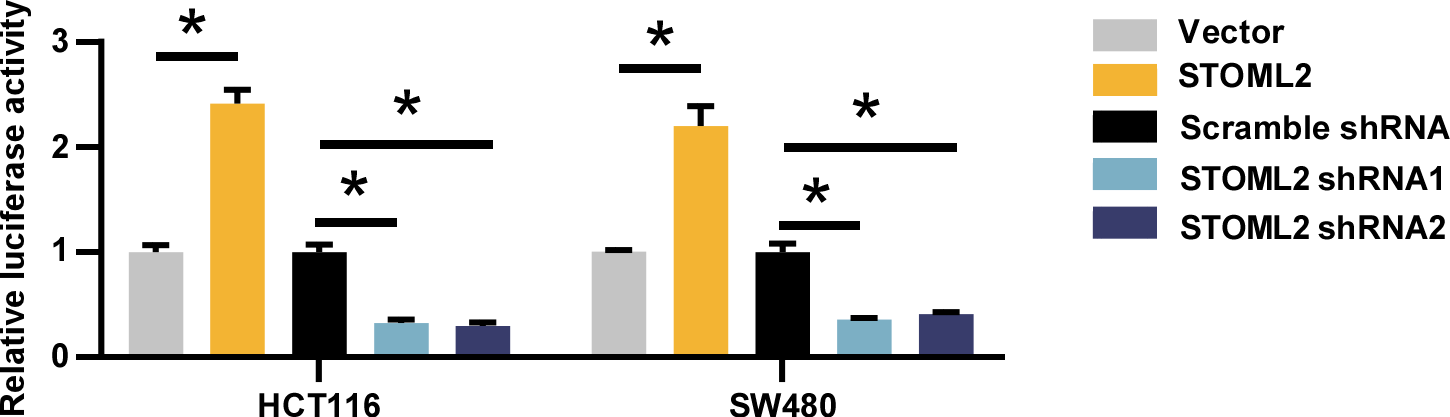
**

Supplemental Figure 2 The luciferase reporter activity of NF-κB was detected in indicated CRC cell lines.

**Supplemental Tables**

**Supplemental Table 1 Clinicopathological characteristics of studied patients and expression of STOML2 in CRC.**

| **Factor** |  | **No.** | **(%)** |
| --- | --- | --- | --- |
| **Gender** | Male | 64 | 53.8 |
|  | Female | 55 | 46.2 |
| **Age (years)** | ≤62 | 61 | 51.3 |
|  | >62 | 58 | 48.7 |
| **Tumer location** | Ascending Colon | 17 | 14.3 |
|  | Transverse Colon | 13 | 10.9 |
|  | Escending Colon | 17 | 14.3 |
|  | Sigmoid Colon | 14 | 11.8 |
|  | Rectal | 58 | 48.7 |
| **Clinical stage** | I | 17 | 14.3 |
|  | II | 33 | 27.7 |
|  | III | 42 | 35.3 |
|  | IV | 27 | 22.7 |
| **T classification** | T_1_ | 2 | 1.7 |
|  | T_2_ | 27 | 22.7 |
|  | T_3_ | 41 | 34.5 |
|  | T_4_ | 49 | 41.2 |
| **N classification** | N_0_ | 59 | 49.6 |
|  | N_1_ | 37 | 31.1 |
|  | N_2_ | 23 | 19.3 |
| **M classification** | M0 | 92 | 77.3 |
|  | M1 | 27 | 22.7 |
| **Histological differentiation** | Well | 34 | 28.6 |
|  | Moderate | 41 | 34.5 |
|  | Poor | 44 | 37.0 |
| **Expression of STOML2** | Low expression | 61 | 51.3 |
|  | High expression | 58 | 48.7 |

**Supplemental Table 2 Real-time polymerase chain reaction (PCR) primers.**

| **Primer name** | **Primer sequence(5′ → 3′)** |
| --- | --- |
| STOML2 | F: GTGACTCTCGACAATGTAAC |
|  | R: TGATCTCATAACGGAGGCAG |
| BCLXL | F: CTCCTCTCCCGACCTGTGAT |
|  | R: AGAGAAAGAGATTCAAATCCGCCT |
| TNF | F: CTGCTGCACTTT GGAGTGAT |
|  | R: AGATGATCTGACTGCCTGGG |
| HIF1A | F: GAAGACATCGCGGGGAC |
|  | R: TGGCTGCATCTCGAGACTTT |
| IL-1B | F: GAAGCTG ATGGCCCTAAACA |
|  | R: AA GCCCTTGCTGTAGTGGTG |
| CCND1 | F: TCCTCTCCAAAATGCCAGAG |
|  | R: GG CGGATTGGAAATGAACTT |
| NFKBIA | F: AAGTGATCCGCCAGGTGAAG |
|  | R: CTGCTCACAGGCAAGGTGTA |
| IL-6 | F: AGTGAGGAACAAGCCAGAGC |
|  | R: GTCAGGGGTGGTTATTGCAT |
| VEGFC | F: CGGACTCGACCTCTCGG |
|  | R: TGGACACAGACCGTAACTGC |
| GAPDH | F: ATTCCACCCATGGCAAA TTC |
|  | R: TGGGATTTCCATTGATGACAAG |

**Supplemental Table 3 Correlation between the clinicopathological features and expression of STOML2 in CRC of female.**

| **Patient characteristics** | | **STOML2 expression** | | ***P*-value** |
| --- | --- | --- | --- | --- |
|  |  | **Low or none** | **High** |  |
| **Age (years)** | ≤63 | 13 | 16 | 0.504 |
|  | >63 | 14 | 12 |  |
| **Tumor location** | ascending colon | 6 | 3 | 0.289 |
|  | transverse colon | 4 | 2 |  |
|  | escending colon | 1 | 4 |  |
|  | Sigmoid colon | 4 | 2 |  |
|  | rectal | 12 | 17 |  |
| **Clinical stage** | I | 3 | 0 | 0.011 |
|  | II | 12 | 4 |  |
|  | III | 8 | 14 |  |
|  | IV | 4 | 10 |  |
| **T classification** | T_1_ | 0 | 0 | 0.185 |
|  | T_2_ | 6 | 3 |  |
|  | T_3_ | 12 | 9 |  |
|  | T_4_ | 9 | 16 |  |
| **N classification** | N_0_ | 18 | 8 | 0.014 |
|  | N_1_ | 6 | 10 |  |
|  | N_2_ | 3 | 10 |  |
| **M classification** | No | 23 | 18 | 0.075 |
|  | Yes | 4 | 10 |  |
| **Histological**  **differentiation** | Well | 9 | 9 | 0.305 |
|  | Moderate | 13 | 9 |  |
|  | Poor | 5 | 10 |  |

**Supplemental Table 4 Correlation between the clinicopathological features and expression of STOML2 in CRC of male.**

| **Patient characteristics** | | **STOML2 expression** | | ***P*-value** |
| --- | --- | --- | --- | --- |
|  |  | **Low or none** | **High** |  |
| **Age (years)** | ≤61 | 19 | 14 | 0.462 |
|  | >61 | 15 | 16 |  |
| **Tumor location** | ascending colon | 5 | 3 | 0.611 |
|  | transverse colon | 5 | 2 |  |
|  | escending colon | 5 | 7 |  |
|  | Sigmoid colon | 3 | 5 |  |
|  | rectal | 16 | 13 |  |
| **Clinical stage** | I | 12 | 2 | 0.049 |
|  | II | 8 | 9 |  |
|  | III | 9 | 11 |  |
|  | IV | 5 | 8 |  |
| **T classification** | T_1_ | 2 | 0 | 0.018 |
|  | T_2_ | 14 | 4 |  |
|  | T_3_ | 10 | 10 |  |
|  | T_4_ | 8 | 16 |  |
| **N classification** | N_0_ | 22 | 11 | 0.081 |
|  | N_1_ | 8 | 13 |  |
|  | N_2_ | 4 | 6 |  |
| **M classification** | No | 29 | 22 | 0.235 |
|  | Yes | 5 | 8 |  |
| **Histological**  **differentiation** | Well | 12 | 4 | 0.019 |
|  | Moderate | 12 | 7 |  |
|  | Poor | 10 | 19 |  |
